# Hidden biodiversity in entomological collections: The overlooked co-occurrence of dipteran and hymenopteran ant parasitoids in stored biological material

**DOI:** 10.1371/journal.pone.0184614

**Published:** 2017-09-19

**Authors:** Gabriela Pérez-Lachaud, Jean-Paul Lachaud

**Affiliations:** 1 Departamento de Conservación de la Biodiversidad, El Colegio de la Frontera Sur, Chetumal, Quintana Roo, Mexico; 2 Centre de Recherches sur la Cognition Animale, Centre de Biologie Intégrative, Université de Toulouse UPS, Toulouse, France; University of Innsbruck, AUSTRIA

## Abstract

Biological collections around the world are the repository of biodiversity on Earth; they also hold a large quantity of unsorted, unidentified, or misidentified material and can house behavioral information on species that are difficult to access or no longer available to science. Among the unsorted, alcohol-preserved material stored in the Formicidae Collection of the ‘El Colegio de la Frontera Sur’ Research Center (Chetumal, Mexico), we found nine colonies of the ponerine ant *Neoponera villosa*, that had been collected in bromeliads at Calakmul (Campeche, Mexico) in 1999. Ants and their brood were revised for the presence of any sign of parasitism. Cocoons were dissected and their content examined under a stereomicroscope. Six *N*. *villosa* prepupae had been attacked by the ectoparasitoid syrphid fly *Hypselosyrphus trigonus* Hull (Syrphidae: Microdontinae), to date the only known dipteran species of the Microdontinae with a parasitoid lifestyle. In addition, six male pupae from three colonies contained gregarious endoparasitoid wasps. These were specialized in parasitizing this specific host caste as no gyne or worker pupae displayed signs of having been attacked. Only immature stages (larvae and pupae) of the wasp could be obtained. Due to the long storage period, DNA amplification failed; however, based on biological and morphological data, pupae were placed in the Encyrtidae family. This is the first record of an encyrtid wasp parasitizing *N*. *villosa*, and the second example of an encyrtid as a primary parasitoid of ants. Furthermore, it is also the first record of co-occurrence of a dipteran ectoparasitoid and a hymenopteran endoparasitoid living in sympatry within the same population of host ants. Our findings highlight the importance of biological collections as reservoirs of hidden biodiversity, not only at the taxonomic level, but also at the behavioral level, revealing complex living networks. They also highlight the need for funding in order to carry out biodiversity inventories and manage existing collections.

## Introduction

Specimens stored in natural history collections, together with their associated data, provide a rich repository of information on biodiversity, ecology, behavior, natural resources, species interactions, and ecosystems [1–3]. With about 3 billion specimens accumulated over 250 years [4,5], collections in natural history museums around the world are the primary archives and physical databases of global species biodiversity [6,7]. For species that are unidentified, difficult to access, extinct in historical times or threatened, these collections also constitute a critical resource and are the only means to access such information and document populations or locations no longer available to science [8–10]. This is clearly evident for endangered species such as primates, as collections can provide data on anatomy, developmental biology, and life history traits that cannot be replicated today, allowing for comparisons between modern and historical environmental and behavioral variables [10]. Information derived from specimens in biological collections has provided the foundation for numerous studies concerning distributional changes over time and habitat modification, or under different climatic conditions [2,11,12]. It has led to applications including species distribution modeling, tracking patterns in phenology and resources use [13–16], or constructing inter-species interactions networks and prediction models with applications in ecology, biodiversity, and emerging diseases [17].

Most museums have a large number of unidentified or misidentified specimens categorized only to a higher taxonomic level, and numerous collections of the most diverse taxa, such as insects, are not yet data based to species level [7]. Natural history collections are thus expected to contain a significant amount of new, yet undescribed taxa [6,7,18]. This situation is especially true for arthropods, which constitute the vast majority of multicellular species on Earth [19–21]. Recent estimations [21–23] suggest that about two-thirds of all arthropod species still await discovery and description. However, it has long been stated that a significant part of this unknown segment of biodiversity was already housed in museum collections as unidentified material [24], and this still appears to be the case. According to Bebber and colleagues [18], a lag of 23–25 years exists between the date a specimen of a new plant species is collected for the first time and when it is subsequently described and published. Similarly, based on a random set of species described in 2007 across all kingdoms, the average shelf life between discovery and description of a new species was also found to be 21 years [6]. Biological collections in Mexico are not the exception and may house an amazing hidden biodiversity, particularly in those taxa that have been collected in association with a surveyed group, and were preserved and stored, but were considered at this time as not deserving any specific study.

Mexico is a major hotspot of biodiversity, supporting at least 10% of the world diversity for many taxa [25]; however, as for many developing countries where biodiversity inventories and collections are a long way down the list of priorities, Mexican biota are only partly inventoried, and consequently there is a pressing need of support by funding agencies and institutions that promote biodiversity studies, along with an efficient management of biological collections. The Arthropod Collection of El Colegio de la Frontera Sur (ECOSUR) in Chetumal, Quintana Roo, Mexico (ECO-CH-AR), was established to house the accumulated biological material that resulted from multiple research projects of various nature [26]. Though some groups (such as Araneae or Lepidoptera) have been organized, identified, and inventoried, other taxa such as Formicidae (Hymenoptera) have been incompletely curated, and many specimens were not organized until 2013. From this date onwards, and as part of a global initiative to strengthen and modernize the biological collections managed by ECOSUR, ants stored in the ECO-CH-AR have been revised, identified, and all associated information digitized, aiming to both constitute a specific “Collection of Formicidae” (ECO-CH-F, see [27]) and make the information available.

During the revision of this material, two previously overlooked parasitoids were found in the cocoons collected in 1999 from various colonies of the arboricolous ponerine ant *Neoponera villosa* and then stored in the ECO-CH-AR: the only syrphid fly known to be a primary parasitoid of ants, an association described very recently from live material [28], but which, stored in alcohol, had been overlooked for 15 years, and an endoparasitoid wasp specialized in attacking only male ant pupae, a remarkable association representing only the second case of primary parasitoidism on ants in the Encyrtidae family [29].

## Materials and methods

### Ethics statement

N/A—All the research was performed on alcohol stored material from a museum collection.

### Ant host

Based on strong molecular and morphological evidence, the Neotropical ant genus *Neoponera* has recently been revived as a distinct genus from *Pachycondyla* [30]. *Neoponera villosa* (Fabricius) is an opportunistic cavity-breeder that nests in dead wood, in cavities at the forks of live branches, in bromeliads, and in abandoned or peripheral cavities of myrmecophytic *Cecropia* spp. [31–34]. Widespread from tropical wet forests to dry scrub forest and even disturbed areas [35], it is found from southern Texas to northern Argentina [34]. Workers are generalized arboreal predators preying on arthropods as well as collecting vegetable matter and liquids such as extra-floral nectar [31,36,37].

Along with 12 other species, *N*. *villosa* belongs to the Neotropical *N*. *foetida* species complex [38,39]. Following the available keys for this group [34,39], workers from our material run to *N*. *villosa* as the anterior face of the petiole is almost straight, vertical, the posterior face broadly convex, the base of the legs reddish (see S1 Fig), and the anterior margin of the clypeus concave medially. We further compared our male and female specimens to the figures illustrating both castes [39]. Until now, only *N*. *villosa* has been reported in the Yucatan Peninsula (with the exception of an unreliable report of *N*. *inversa* in Yucatan [40]), and it is the only species within the *N*. *foetida* species complex to have been reported inhabiting epiphytes such as *Aechmea bracteata* [33,41]. In order to discard any confusion between *N*. *villosa*, *N*. *curvinodis*, and *N*. *inversa*–all three species belonging to the *N*. *foetida* species complex and reported in southern Mexico– a sample of workers from our host species was sent to the specialist of this group, JHC Delabie (Laboratório de Mirmecologia, Itabuna, Bahia, Brazil), who confirmed the identity of our species as *N*. *villosa*.

### Sample data and revision

Unsorted, alcohol-preserved material corresponding to relatively complete nests of *N*. *villosa* (with both adults and brood present) was stored in the ECO-CH-AR collection. The original material was gathered during research on the diversity of frogs in bromeliads close to the town of Zoh-Laguna, Calakmul, Campeche, Mexico (18°35' N, 89°26' W, 270 m above sea level), at the eastern side of the Calakmul Biosphere Reserve [42]. In this study, 60 individual tank bromeliads (*Aechmea bracteata* (Swartz) Grisebach) were sampled for frogs at the end of the dry season, 21–24 April 1999. Almost half of the bromeliads were collected in seasonally flooded forest.

Nine samples with adult ants and brood were obtained, corresponding to the material collected from nine different bromeliads (see S1 Table for exact localition and colony sample composition). Brood and adults numbers, sex, and caste were all recorded. Adults and larvae were examined for the presence of any external sign of parasitism (i.e. possible scars, alien larvae attached to the cuticle, visible external changes, or respiratory funnels, see [29,43]); cocoons were dissected and their content examined under a stereomicroscope (Nikon SMZ745T, magnification 6.5 to 50x). Dissection of cocoons yielded several fly larvae and puparia, in addition to a number of wasp larvae and pupae (see Results). Voucher specimens of both parasitoids and ants were deposited in the arthropod (ECO-CH-AR: HV-044–049, D-0046, D-0047) and formicid (ECO-CH-F: F-0275, F-0276, F-0281, F-0287, F-0301, F-0303, F-308, F-0310, F-0316) collections of El Colegio de la Frontera Sur in Chetumal (Quintana Roo, Mexico).

### Parasitoid identification

Fly larvae and puparia were compared with voucher specimens of similar material that was recently found in *N*. *villosa* nests at Kohunlich (Quintana Roo, Mexico) [28] and conserved in the ECO-CH-AR collection.

Wasp pupae morphologically corresponded to a member of the Chalcidoidea, but the early stage of development of these pupae precluded their identification beyond the family level. Several techniques were used to extract and amplify the wasp DNA from pupae obtained from dissected hosts: standardized extraction protocols of the BOLD initiative [44], using the Phire^™^ Tissue Direct PCR master Mix #F-170S kit (Thermo Scientific Baltics UAB, Vilnius, Lithuania) [45], or using Chelex^®^ 100 (Bio-Rad, Hercules, CA, USA) [46]. Additionally, we used family-level taxonomic keys to differentiate pupae [47], and the material was compared to voucher specimens of pupae of *Blanchardiscus pollux*, the only encyrtid wasp known to date as a primary parasitoid of ants [29] and conserved in the ECO-CH-AR collection.

## Results

Six of the nine colony samples (four queenright and two queenless colonies) were almost complete and contained numerous adults and brood; three other vials contained only a few workers and almost no cocoons (S1 Table). The six almost complete colony samples presented between 0 and 5 dealate females, 85.8 ± 24.2 workers (mean ± SEM, range: 36–197), and 18.0 ± 4.1 cocoons (range 7–32). Alate females and males were present in only four and three colonies, respectively.

A total of 759 adults, 112 cocoons, and 12 larvae were examined (S1 Table). Four out of the nine colony samples were parasitized. None of the larvae and adult individuals showed signs of parasitism; in contrast, 12 cocoons (10.7%) were parasitized. Dissections demonstrated that these cocoons had been attacked by two different parasitoid species (Table 1).

**Table 1:** Data obtained from the dissection of the cocoons of all the *Neoponera villosa* colony samples containing brood. Figures correspond to the numbers of immature stages present, dissected cocoons, parasitized and non-parasitized cocoons for each host caste, and to the parasitism rates and the identity of the parasitoids.

ECO-CH-F Code	Larvae	Cocoons	Cocoon dissection				Non-parasitized cocoons	Parasitized cocoons	% parasitism	Parasitoid identity	Observations
			Prepupae	Male pupae	Gyne pupae	Worker pupae					
F-0275	7	0	-	-	-	-	-	-	NA		
F-0276	0	2	2	0	0	0	2	0	0		
F-0281	2	23	5	14	0	4	20	3	13.0	Encyrtidae sp.	3 *N*. *villosa* male pupae parasitized^a^^,^^b^
F-0287	1	32	14	2	10	6	30	2	6.3	Encyrtidae sp.	2 *N*. *villosa* male pupae parasitized^c^
F-0301	0	8	2	6	0	0	7	1	12.5	Encyrtidae sp.	1 *N*. *villosa* male pupa parasitized^d^
F-0303	2	25	15	7	1	2	25	0	0		
F-0308	0	7	1	0	0	6	1	6	85.7	*Hypselosyrphus trigonus*	3 L1/prepuae, 1 L3, 2 puparia
F-0310	0	13	6	0	0	7	13	0	0		
F-0316	0	2	2	0	0	0	2	0	0		
Total^e^	12	112	47	29	11	25	100	12	10.7		

^a^: Parasitized male ant pupae presented very reduced antennae and showed some deformation of the legs.

^b^: One host pupa dissected yielded 21 parasitoids (20 pupae, 1 larva); the second pupa contained parasitoid larvae and the third contained several parasitoid pupae; however, the latter two host pupae were not completely dissected to preserve them as voucher specimens.

^c^: Both host pupae not completely dissected to preserve them as voucher specimens; the first contained parasitoid larvae, the second parasitoid pupae.

^d^: Host pupa dissected; dissection yielded 43 parasitoid pupae.

^e^: Except for “% parasitism” that corresponds to the mean.

**Fig 1:**
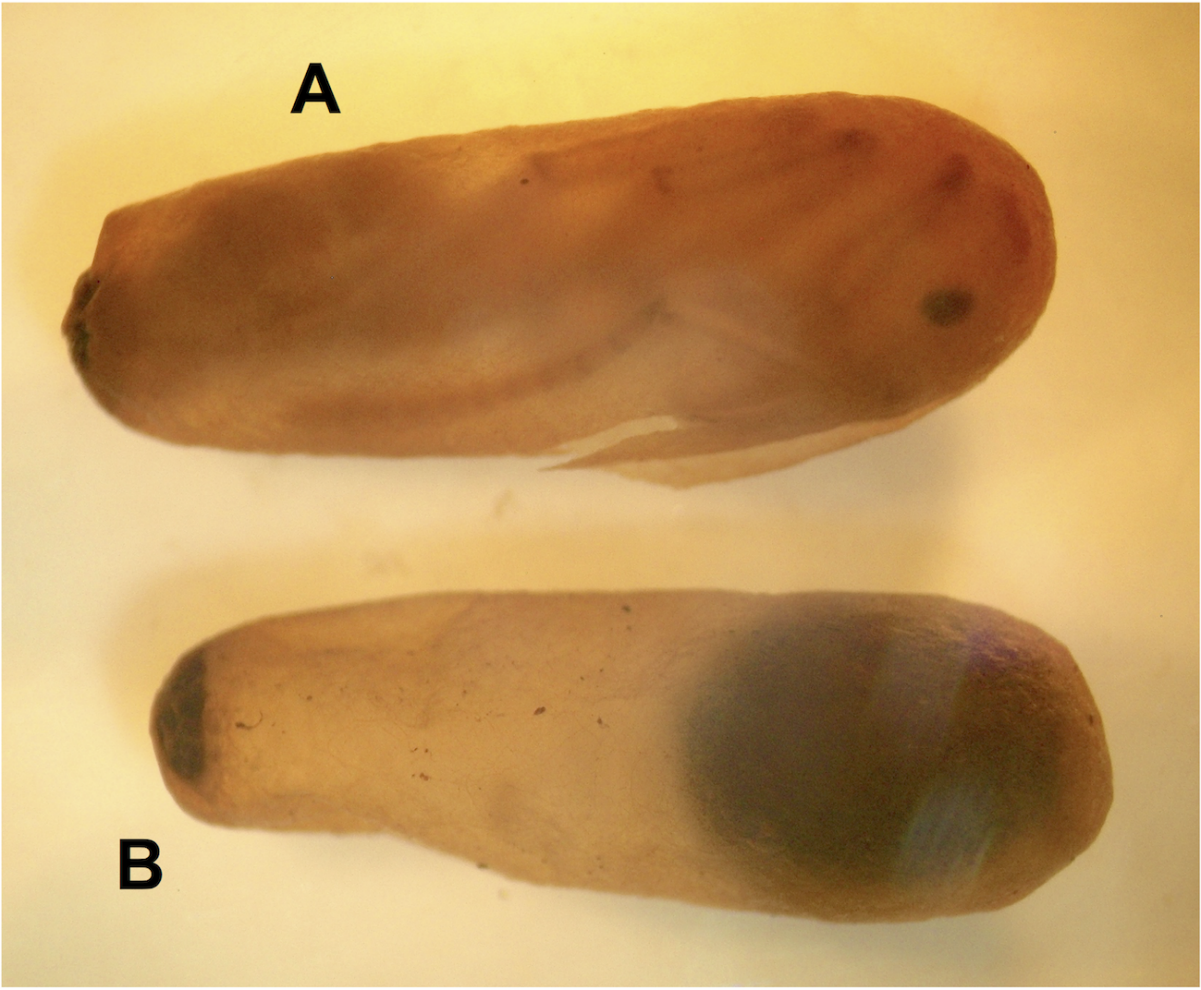
General aspect of alcohol preserved cocoons of *Neoponera villosa*. (A) Cocoon containing a worker pupa. (B) Another cocoon, containing a puparium of the ectoparasitoid syrphid fly *Hypselosyrphus trigonus*. Photo: G. Pérez-Lachaud.

Six cocoons, all belonging to a single colony, contained worker ant prepupae (or their remains) that have been attacked by the syrphid fly *Hypselosyrphus trigonus*, a solitary ectoparasitoid of the host prepupae that develops within the protection of the cocoon’s silky envelope (Fig 1). Three first instar larvae, one last instar larva, and two puparia of this parasitoid fly were secured. Only one cocoon out of the seven found in this colony sample resulted unparasitized (Table 1).

**Fig 2:**
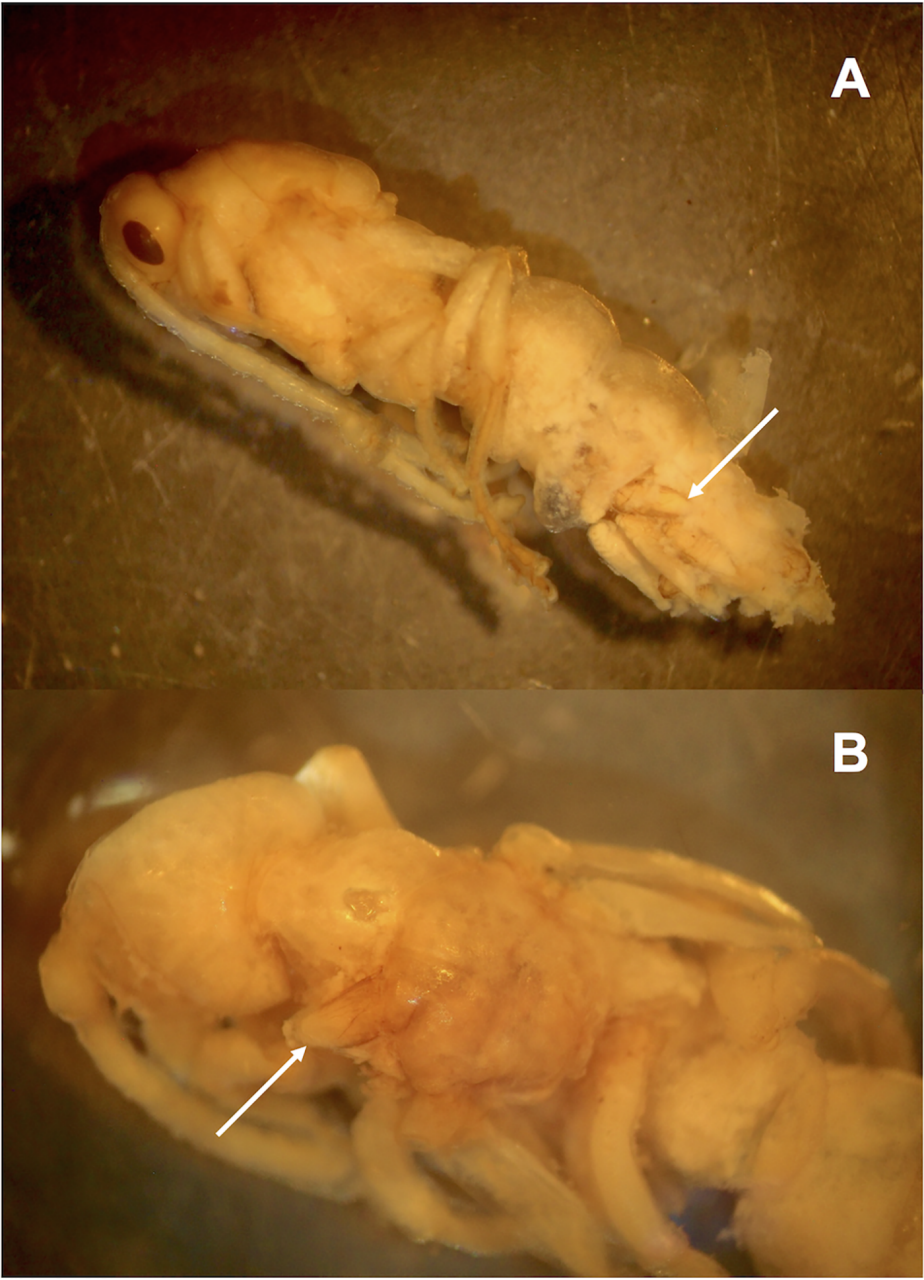
*Neoponera villosa* male pupa parasitized by a gregarious hymnopteran endoparasitoid. The parasitoid wasp larvae and pupae occupy both the abdomen (A) and the thorax (B) of the ant pupa (arrows indicate the developing parasitoids). Photos: G. Pérez-Lachaud.

**Fig 3:**
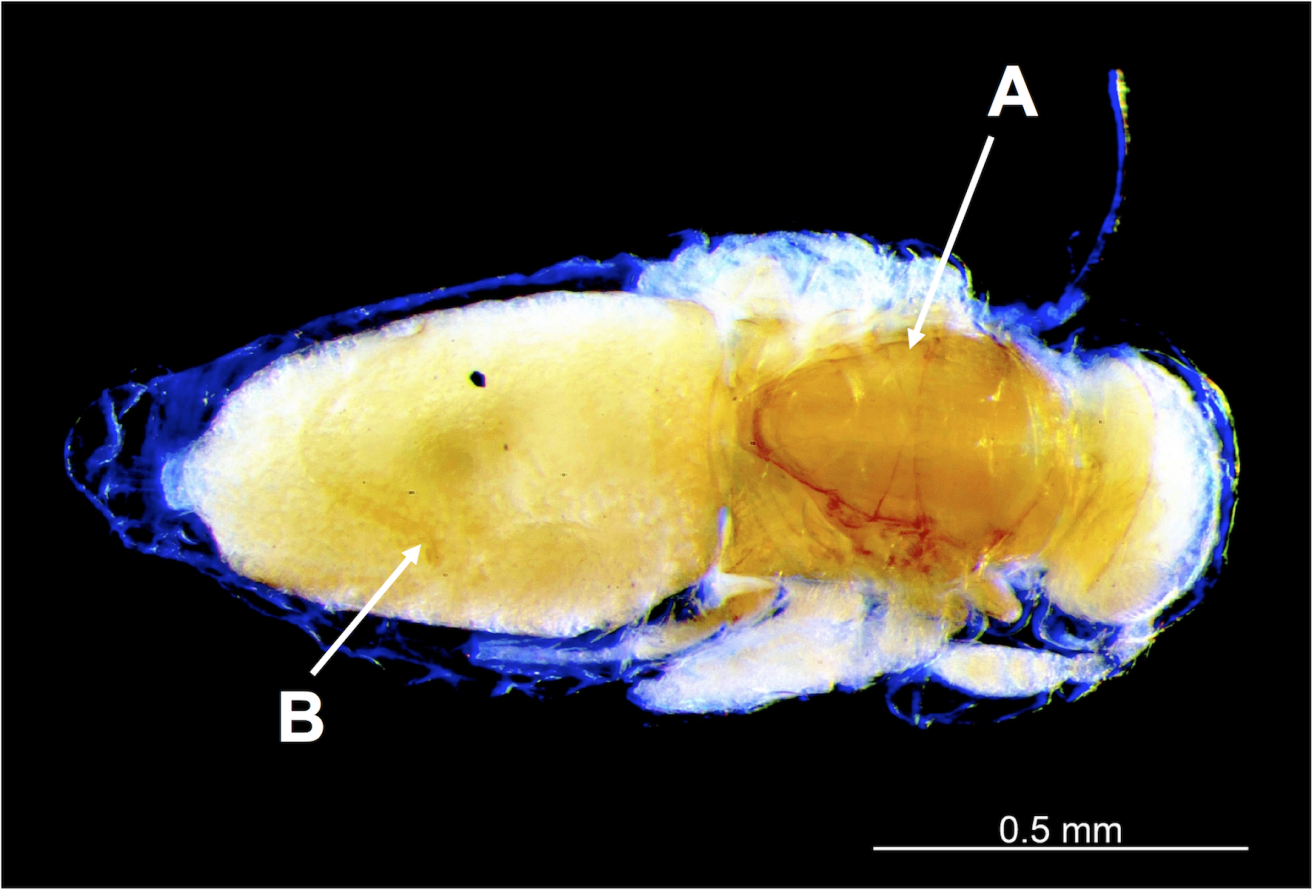
Unidentified encyrtid wasp pupa found within the abdomen of a *Neoponera villosa* male pupa. Arrows indicate two of the diagnostic characters of this family: (A) the axillae are acutely transverse-triangular, meeting medially; (B) the metasoma presents cerci advanced anteriorly. Photo: H. Bahena Basave and G. Pérez-Lachaud.

**Fig 4:**
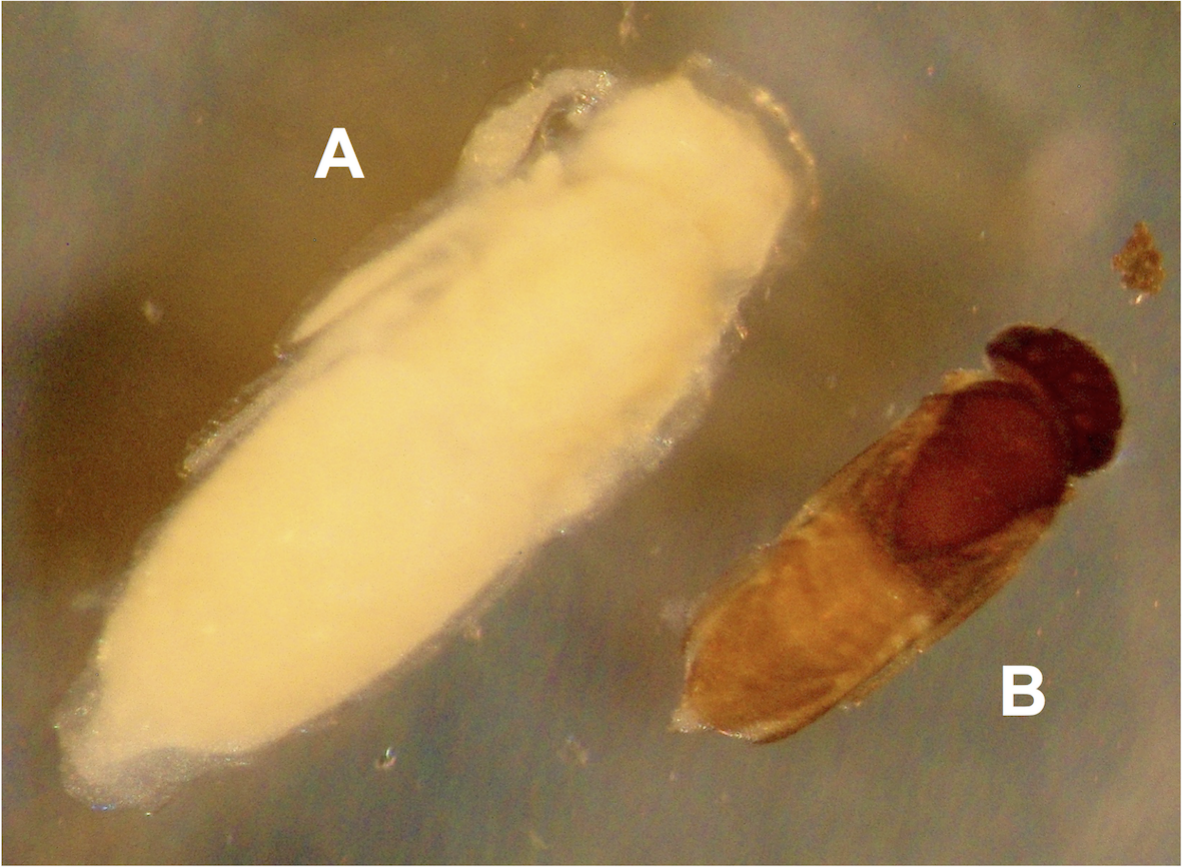
The only two ant endoparasitoids known among encyrtid wasps. (A) Pupa of the unidentified encyrtid wasp parasitizing *Neoponera villosa* in Mexico. (B) The encyrtid wasp *Blanchardiscus pollux* parasitizing *Neoponera goeldii* in French Guiana. Photo: G. Pérez-Lachaud.

Six other cocoons, belonging to three different colonies, were parasitized by a gregarious endoparasitoid wasp. Though male, gyne, and worker pupae were available as potential hosts, the attacks significantly targeted male pupae (see Table 1): 6/29 male pupae parasitized, 0/11 gyne pupae, 0/25 worker pupae, and 0/47 unidentified prepupae (Fisher’s exact test for count data, two-sided p = 0.001147). Numerous parasitoid wasp larvae or pupae were present inside each parasitized host pupa, grouped in dense clusters at the level of the abdomen (Fig 2A) and thorax (Fig 2B) of the already formed ant male pupa. Up to 43 parasitoid wasp pupae were found in a single host pupa. Though several protocols of DNA extraction and amplification were assayed (see Material and methods), we failed to acquire sufficient DNA to obtain a sequence, and molecular identification was not possible. However, the position and general aspect of the developing parasitoids inside the ant host body strongly resembled that of the larvae and pupae of the encyrtid wasp *Blanchardiscus pollux* when parasitizing pupae of the congeneric arboricolous ant *N*. *goeldii* [29], thus strongly suggesting that the hymenopteran larvae and pupae in our study belong to the Encyrtidae family. This is further supported by various morphological traits: the mesocoxae are inserted anterior to midline of mesopleuron, much closer to precoxae than to metacoxae, the axillae are acutely transverse-triangular, meeting medially (Fig 3A), and the cerci on the metasoma are advanced anteriorly (Fig 3B); all traits conforming the diagnostic characters of this family [47]. Wing venation was the only other character defining Encyrtidae that could not be assessed on our pupae. The parasitoid pupae associated with *N*. *villosa* were significantly larger than those of *B*. *pollux* (Fig 4), strongly suggesting a different species at least.

## Discussion

Each year, taxon experts name and describe about 15,000–18,000 new species in addition to improving our understanding of already known species [7,22]. New species are identified on the basis of new collection effort and through discriminating among cryptic species or previously “hidden” material stored in natural museum collections. Beyond their extensive use for taxonomic and phylogenetic research, scientific museum collections are also becoming critical sources of information in a wide range of fields, from biotic communities interaction and structure to environmental monitoring and human health [1,2,48]. Our findings represent an example of the potential of biological collections to house behavioral information in addition to taxonomic diversity. We report on the discovery of a new hymenopteran primary parasitoid of ants from *N*. *villosa* colonies that had been stored in alcohol without detection for the past 18 years. Our research also confirms the attack by another primary ant parasitoid, the syrphid fly *H*. *trigonus*. Both endoparasitoid hymenopteran and ectoparasitoid dipteran species co-occurred in the same ant host population.

Thousands of myrmecophilous invertebrates have been inventoried to date [49–54] although, with regard to ant parasitoids, there is still a noteworthy lack of knowledge despite some recent efforts endeavored to reduce this gap [55,56]. Insect parasitoid species that attack ant immature stages or adults are present in nine families belonging to the order Hymenoptera [29,55,56] and four families from the order Diptera [28,57–59]. During the last five years, three of these families, Encyrtidae for hymenopterans and Syrphidae and Chloropidae for dipterans, have been reported for the first time as primary ant parasitoids, a clear indication that, to improve our knowledge on ant parasitoids, a more sustained sampling effort is required.

Many undiscovered species are difficult to encounter because they are cryptic, small in size, or nesting in microhabitats with low accessibility [60,61]; furthermore, many new taxa with elusive life histories are destined to remain undiscovered, even in the best-studied parts of the world [20]. Our results suggest that although many more ant parasitoids certainly await discovery in the field, a large number of them, possibly small size endoparasitoids such as the encyrtid wasps reported here, are potentially available to science, but inadvertenly hidden in collections all around the world.

Most encyrtids are endoparasitoids of arachnids and insects, or hyperparasitoids of other hymenopteran parasitoids, and almost half of all the species for which the host is known are parasitic upon Coccoidea [62]. Originating from the unsorted material of the ECO-CH-AR collection, our discovery of an encyrtid wasp parasitizing *N*. *villosa* pupae is only the second record of an encyrtid parasitizing ants. It is noteworthy that the first report of a gregarious endoparasitoid encyrtid wasp (*B*. *polux*) parasitizing ants was also fortuitously found by revising material that had been stored for ten years of the arboreal ant *N*. *goeldii* from French Guiana [29]. While *B*. *pollux* parasitized both worker and gyne pupae of *N*. *goeldii*, the unidentified encyrtid associated with *N*. *villosa* revealed a clear host caste specificity by attacking only male pupae. Though the low rate of parasitism might seem insignificant at the host ant population level, the fact that in the present case sexual forms are the main targets of parasitism suggests that encyrtid parasitoids may exact a significant fitness cost to their ant hosts. Very few cases of parasitoids attacking exclusively sexual castes of their ant host have been reported from diapriid and eurytomid wasps [55] or from phorid flies [45, 63].

*Hypselosyrphus trigonus* is a neotropical microdontine fly species resembling a stingless bee. Described by Hull [64] on the basis of a single individual, its parasitic lifestyle on *N*. *villosa* prepupae has been reported only very recently [28], based on rearing data. Information on dipteran parasitoids of ants has been recently reviewed [45]. Originally described using specimens from Barro Colorado, Panama [64], *H*. *trigonus* has been reported in Belize and in the Mexican state of Quintana Roo [28]. Our research extends its known distribution to the Mexican state of Campeche in association with *N*. *villosa* nesting in *Aechmea* bromeliads.

Considering the extended distribution of *N*. *villosa* in America, it remains to be studied whether or not this species is parasitized by both parasitoid species throughout its distribution range. According to predictions formulated by the geographical mosaic model [65], geographical variation in the strengths of coevolution may lead to differences in ant-host use at both local and regional levels. Thus, in some cases, not only the availability of host species, but also the presence of other competitors might shape coevolution between particular species of social parasites and their hosts [54]. Field collecting in proximity to the sites where our samples were originally collected did not yield more material. In fact, during the last five years, 43 nests of *N*. *villosa* have been collected in different localities of the Yucatan Peninsula (10 of them near the original 1999 site) ([28] and Rocha, Lachaud, and Pérez-Lachaud, unpub. data) confirming the presence of the syrphid fly *H*. *trigonus* in some localities; however, not one single encyrtid wasp has been found again to allow the isolation of high-quality DNA.

Our results emphasize that examining insect material already housed in collections will certainly result in the discovery of new species to science and will unveil unanticipated biological interactions and complex living networks. Identified species of tropical arthropods make the largest contribution to overall global species richness [22], and many more species are expected to be discovered at diversity hotspots [61,66], the very areas that are most threatened by anthropogenic and global changes [20,60]. Approximately 80% of extant species remain to be described [23,67], and it is likely that a large proportion of these species may become extinct over the next few decades. A management of natural history collections that is focused on the careful scrutiny of stored material, combined with new generation molecular sequencing, will be central to the successful exploration of species diversity. This would most likely reveal unexpected biodiversity, adding critically important data for species identification, phylogenetic reconstructions, and interaction networks. Adequate funding that allows both biodiversity inventories and proper curatorial management of existing collections will be critical in achieving such an urgent goal.

## Supporting information

S1 Table *Neoponera villosa* colony samples used in this study. All colony samples were collected in 1999 from Zoh-Laguna, Calakmul (Campeche, Mexico) and stored in the Formicidae Collection (ECO-CH-F) of El Colegio de la Frontera Sur in Chetumal (Quintana Roo, Mexico).
(PDF)

S1 Fig A worker of the ant host *Neoponera villosa*. (A) Left profile of the worker showing the almost straight, vertical anterior face of the petiole, the posterior face broadly convex, and the reddish base of the legs. (B) Close-up of the petiole. Photo: J-P Lachaud.
(PDF)

## References

[1] Suarez AV, Tsutsui ND. The value of museum collections for research and society. BioScience. 2004; 54: 66–74.

[2] Pyke GH, Ehrlich PR. Biological collections and ecological/environmental research: a review, some observations and a look to the future. Biological Reviews. 2010; 85: 247–266. doi: 10.1111/j.1469-185X.2009.00098.x 19961469 10.1111/j.1469-185X.2009.00098.x

[3] Hill A, Guralnick R, Smith A, Sallans A, Gillespie R, Denslow M, et al The notes from nature tool for unlocking biodiversity records from museum records through citizen science. ZooKeys. 2012;209: 219–233. 10.3897/zookeys.209.3472 PMC3406478 22859890

[4] Ariño AH. Approaches to estimating the universe of natural hitory collections data. Biodiv Inform. 2010;7: 81–92.

[5] Smith VS, Blagoderov V. Bringing collections out of the dark. ZooKeys. 2012;209: 1–6. 10.3897/zookeys.209.3699 PMC3406462 22859874

[6] Fontaine B, Perrard A, Bouchet P. 21 years of shelf life between discovery and description of new species. Curr Biol. 2012;22: R943–R944. doi: 10.1016/j.cub.2012.10.029 23174292 10.1016/j.cub.2012.10.029

[7] Wheeler QD, Knapp S, Stevenson DW, Stevenson J, Blum SD, Boom BM, et al Mapping the biosphere: exploring species to understand the origin, organization and sustainability of biodiversity. Syst Biodiv. 2012;10: 1–20.

[8] Ricklefs RE. Old specimens and new directions: the museum tradition in contemporary ornithology. Auk. 1980; 97: 206–207.

[9] D’Elia J, Haig SM, Mullins TD, Miller MP. Ancient DNA reveals substantial genetic diversity in the *California Condor* (Gymnogyps californiacus) prior to a population bottleneck. Condor. 2016; 118: 703–714.

[10] Aronsen GP, Kirkham M. Inventory and assessment of the *Pan troglodytes* (Blumenbach, 1799) skeletal collection housed at the Yale Peabody Museum. Bulletin of the Peabody Museum of Natural History. 2017; 58: 209–259.

[11] Polus E, Vandewoestijne S, Choutt J, Baguette M. Tracking the effects of one century of habitat loss and fragmentation on calcareous grassland butterfly communities. Biodivers Conserv. 2007; 16: 3423–3436.

[12] Brooks SJ, Self A, Toloni F, Sparks T. Natural history museum collections provide information on phenological change in British butterflies since the late-nineteenth century. International Journal of Biometeorology. 2014; 58: 1749–1758. doi: 10.1007/s00484-013-0780-6 24429705 10.1007/s00484-013-0780-6

[13] Harper GL, Maclean N, Goulson D. Analysis of museum specimens suggests extreme genetic drift in the adonis blue butterfly (*Polyommatus bellargus*). Biological Journal of the Linnean Society. 2006; 88: 447–452.

[14] Giovanelli JGR, Haddad CFB, Alexandrino J. Predicting the potential distribution of the alien invasive American bullfrog (*Lithobates catesbeianus*) in Brazil. Biol Invasions. 2008;10: 585–590.

[15] Johnson KG, Brooks SJ, Fenberg PB, Glover AG, James KE, Lister AM, et al Climate change and biosphere response: unlocking the collections vault. BioScience. 2011; 61: 147–153.

[16] Scheper J, Reemer M, van Kats R, Ozinga WA, van der Linden GTJ, Schaminée JHJ, et al Museum specimens reveal loss of pollen host plants as key factor driving wild bee decline in The Netherlands. Proc Natl Acad Sci USA. 2010;107: 22169–22171. 25422416 10.1073/pnas.1412973111 PMC4267333

[17] Stephens CR, Heau JG, González C, Ibarra-Cerdeña CN, Sánchez-Cordero V, González-Salazar C. Using biotic interaction networks for prediction in biodiversity and emerging diseases. PLos ONE. 2009; 4: e5725 doi: 10.1371/journal.pone.0005725 19478956 10.1371/journal.pone.0005725 PMC2685974

[18] Bebber DP, Carine MA, Wood JRI, Wortley AH, Harris D, Prance GT, et al Herbaria are a major frontier for species discovery. Proc Natl Acad Sci USA. 2010;107: 22169–22171. doi: 10.1073/pnas.1011841108 21135225 10.1073/pnas.1011841108 PMC3009773

[19] Basset Y, Cizek L, Cuénoud P, Didham RK, Guilhaumon F, Missa O, et al Arthropod diversity in a tropical forest. Science. 2012;338: 1481–1484. doi: 10.1126/science.1226727 23239740 10.1126/science.1226727

[20] Scheffers BR, Joppa LN, Pimm SL, Laurance WF. What we know and don’t know about Earth’s missing biodiversity. Trends Ecol Evol. 2012;27: 501–510. doi: 10.1016/j.tree.2012.05.008 22784409 10.1016/j.tree.2012.05.008

[21] Stork NE, McBroom J, Gely C, Hamilton AJ. New approaches narrow global species estimates for beetles, insects, and terrestrial arthropods. Proc Natl Acad Sci USA. 2015;112: 7519–7523. doi: 10.1073/pnas.1502408112 26034274 10.1073/pnas.1502408112 PMC4475949

[22] May RM. Tropical arthropod species, more or less? Science. 2010;329: 41–42. doi: 10.1126/science.1191058 20595603 10.1126/science.1191058

[23] Mora C, Tittensor DP, Adl S, Simpson AGB, Worm B. How many species are there on earth and in the ocean? PLoS Biol. 2011;9: e1001127 doi: 10.1371/journal.pbio.1001127 21886479 10.1371/journal.pbio.1001127 PMC3160336

[24] Green SV. The taxonomic impediment in orthopteran research and conservation. J Insect Conserv. 1998;2: 151–159.

[25] Sarukhán J, Dirzo R. Biodiversity-rich countries In: Levin SA, editor. Encyclopedia of biodiversity, Vol 1 San Diego: Academic Press; 2001 pp. 419–436.

[26] Salas Suárez N, Pozo C. Colección de Artrópodos In: León-Cortés JL, Lorenzo Monterrubio C, Pozo C, editors. Colecciones biológicas de El Colegio de la Frontera Sur, México. San Cristobal de las Casas: ECOSUR-CONABIO, Ed.; 2003 pp. 47–57.

[27] Lachaud J-P, Pérez-Lachaud G. Revisión preliminar de las hormigas de Campeche y Quintana Roo, México, con base en la colección de Arthropoda del Colegio de la Frontera Sur In: Vásquez-Bolaños M, Castaño-Meneses G, Cisneros-Caballero A, Quiroz-Rocha GA, Navarrete-Heredia JL, editors. Formicidae de México. Guadalajara: Cuerpo Académico de Zoología UDG-CA-51; 2013 pp. 23–32.

[28] Pérez-Lachaud G, Jervis MA, Reemer M, Lachaud J-P. An unusual, but not unexpected, evolutionary step taken by syrphid flies: the first record of true primary parasitoidism of ants by Microdontinae. Biol J Linn Soc. 2014;111: 462–472.

[29] Pérez-Lachaud G, Noyes J, Lachaud J-P First record of an encyrtid wasp (Hymenoptera: Chalcidoidea) as a true primary parasitoid of ants (Hymenoptera: Formicidae). Fla Entomol. 2012;95: 1066–1076.

[30] Schmidt CA, Shattuck SO. The higher classification of the ant subfamily Ponerinae (Hymenoptera: Formicidae), with a review of ponerine ecology and behavior. Zootaxa. 2014;3817: 1–242. doi: 10.11646/zootaxa.3817.1.1 24943802 10.11646/zootaxa.3817.1.1

[31] Lachaud J-P, Fresneau D, García-Pérez J. Étude des stratégies d'approvisionnement chez 3 espèces de fourmis ponérines (Hymenoptera, Formicidae). Folia Entomol Mex. 1984;61: 159–177.

[32] Pérez-Bautista M, Lachaud J-P, Fresneau D. La división del trabajo en la hormiga primitiva *Neoponera villosa* (Hymenoptera: Formicidae). Folia Entomol Mex. 1985;65: 119–130.

[33] Dejean A, Olmsted I. Ecological studies on *Aechmea bracteata* (Swartz) (Bromeliaceae). J Nat Hist. 1997;31: 1313–1334.

[34] Mackay WP, Mackay EE. The systematics and biology of the New World ants of the genus *Pachycondyla* (Hymenoptera: Formicidae). Lewiston: Edwin Mellon Press; 2010.

[35] Wild AL. The genus *Pachycondyl*a (Hymenoptera: Formicidae) in Paraguay. Bol Mus Nac Hist Nat Parag. 2002;14: 1–18.

[36] Dejean A, Corbara B. Predatory behavior of a Neotropical arboricolous ant: *Pachycondyla villosa* (Formicidae: Ponerinae). Sociobiology. 1990;17: 271–286.

[37] Valenzuela-González J, López-Méndez JA, García-Ballinas A. Ciclo de actividad y aprovisionamiento de *Pachycondyla villosa* (Hymenoptera, Formicidae) en agroecosistemas cacaoteros del Soconusco, Chiapas, México. Folia Entomol Mex. 1994; 91: 9–21.

[38] Lucas C, Fresneau D, Kolmer K, Heinze J, Delabie JHC, Pho DB. A multidisciplinary approach to discriminating different taxa in the species complex *Pachycondyla villosa* (Formicidae). Biol J Linn Soc. 2002;75: 249–259.

[39] Fernandes IO, De Oliveira ML, Delabie JHC. Description of two new species in the Neotropical *Pachycondyla foetida* complex (Hymenoptera: Formicidae: Ponerinae) and taxonomic notes on the genus. Myrmecol News. 2014;19: 133–163.

[40] Kempf WW. Catálogo abreviado das formigas da região neotropical (Hymenoptera: Formicidae). Stud Entomol. 1972; 15: 1–344.

[41] Dejean A, Olmsted I, Snelling RR. Tree-epiphyte-ant relationships in the low inundated forest of Sian Ka’an Biosphere Reserve, Quintana Roo, Mexico. Biotropica. 1995; 27: 57–70.

[42] Galindo-Leal C, Cedeño-Vázquez JR, Calderón R, Augustine J. Arboreal frogs, tank bromeliads and disturbed seasonal tropical forest. Contemp Herpetol. 2003;2003: 1–14.

[43] Pérez-Lachaud G, Heraty JM, Carmichael A, Lachaud J-P. Biology and behavior of *Kapala* (Hymenoptera: Eucharitidae) attacking *Ectatomma*, *Gnamptogenys* and *Pachycondyla* (Formicidae: Ectatomminae and Ponerinae) in Chiapas, Mexico. Ann Entomol Soc Am. 2006;99: 567–576.

[44] Hajibabaei M, deWaard JR, Ivanova NV, Ratnasingham S, Dooh RT, Kirk SL, et al Critical factors for assembling a high volume of DNA barcodes. Phil Trans R Soc B. 2005;360: 1959–1967. doi: 10.1098/rstb.2005.1727 16214753 10.1098/rstb.2005.1727 PMC1609220

[45] Pérez-Lachaud G, Jahyny BJB, Ståhls G, Rotheray G, Delabie JHC, Lachaud J-P Rediscovery and reclassification of the dipteran taxon *Nothomicrodon* Wheeler, an exclusive endoparasitoid of gyne ant larvae. Sci Rep. 2017;7: 45530 doi: 10.1038/srep45530 28361946 10.1038/srep45530 PMC5374537

[46] Folmer O, Black M, Hoeh W, Lutz R, Vrijenhoek R. DNA primers for amplification of mitochondrial cytochrome c oxidase subunit I from diverse metazoan invertebrates. Mol Mar Biol Biotechnol. 1994;3: 294–299. 7881515

[47] Grissell EE, Schauff ME. Superfamily Chalcidoidea In: Gibson GAP, Huber JT, Woolley JB, editors. Annotated keys to the genera of Nearctic Chalcidoidea (Hymenoptera). Ottawa: NRC-CNRC Publication; 1997 pp. 45–116.

[48] DiEuliis D, Johnson KR, Morse SS, Schindel DE. Specimen collections should have a much bigger role in infectious disease research and response. Proc Natl Acad Sci USA. 2016;113: 4–7. doi: 10.1073/pnas.1522680112 26733667 10.1073/pnas.1522680112 PMC4711881

[49] Donisthorpe HStJK. The guests of British ants, Their habits and life histories. London: G. Routledge & Sons Ltd; 1927.

[50] Hölldobler B, Wilson EO. The Ants. Berlin: Springer-Verlag; 1990.

[51] Kistner DH. The social insects’ bestiary In: Hermann HR, editor. Social Insects, Vol 3 New York: Academic Press; 1982 pp. 1–244.

[52] Rettenmeyer CW, Rettenmeyer ME, Joseph J, Berghoff SM. The largest animal association centered on one species: the army ant *Eciton burchellii* and its more than 300 associates. Insect Soc. 2011;58: 281–292.

[53] Parmentier T, Dekoninck W, Wenseleers T. A highly diverse microcosm in a hostile world: a review on the associates of red wood ants (*Formica rufa* group). Insect Soc. 2014;61: 229–237.

[54] Witek M, Barbero F, Markó B. *Myrmica* ants host highly diverse parasitic communities: from social parasites to microbes. Insect Soc. 2014;61: 307–323.

[55] Lachaud J-P, Pérez-Lachaud G. Diversity of species and behavior of hymenopteran parasitoids of ants: a review. Psyche. 2012;Article ID 134746: 24 pp.

[56] Lachaud J-P, Pérez-Lachaud G. Ectaheteromorph ants also host highly diverse parasitic communities: a review of parasitoids of the Neotropical genus *Ectatomma*. Insect Soc. 2015;62: 121–132.

[57] Gösswald K. Pflege des Ameisenparasiten *Tamiclea globula* Meig. (Dipt.) durch den Wirt mit Bemerkungen über den Stoffwechsel in der parasitierten Ameise. Verhandl Deutsch Zool. 1950;1949: 256–264.

[58] Feener DH Jr, Brown BV. Diptera as parasitoids. Annu Rev Entomol. 1997;42: 73–97. doi: 10.1146/annurev.ento.42.1.73 15012308 10.1146/annurev.ento.42.1.73

[59] González CT, Wcislo WT, Cambra R, Wheeler TA, Fernández-Marín H. A bew ectoparasitoid species of *Pseudogaurax* Malloch, 1915 (Diptera: Chloropidae), attacking the fungus-growin g ant, *Apterostigma* dentigerum Wheeler, 1925 (Hymenoptera: Formicidae). Ann Entomol Soc Am 2016;109: 639–645.

[60] Guénard B, Weiser MD, Dunn RR. Global models of ant diversity suggest regions where new discoveries are most likely are under disproportionate deforestation threat. Proc Natl Acad Sci USA. 2012;109: 7368–7373. doi: 10.1073/pnas.1113867109 22529355 10.1073/pnas.1113867109 PMC3358832

[61] Pérez-Lachaud G, Lachaud J-P. Arboreal ant colonies as ‘hot-points’ of cryptic diversity for myrmecophiles: the weaver ant *Camponotus* sp. aff. *textor* and its interaction network with its associates. PLoS ONE. 2014;9:e100155 doi: 10.1371/journal.pone.0100155 24941047 10.1371/journal.pone.0100155 PMC4062527

[62] Noyes JS. Universal Chalcidoidea Database. World Wide Web electronic publication. http://www.nhm.ac.uk/chalcidoids. 2015.

[63] Wojcik DP, Jouvenaz DP, Lofgren CS. First report of a parasitic fly (Diptera: Phoridae) from a red imported fire ant (*Solenopsis* invicta) alate female (Hymenoptera: Formicidae). Fla Entomol. 1987;70: 181–182.

[64] Hull FM. New species of exotic syrphid flies. Psyche. 1937;44: 12–32.

[65] Thompson JN. Specific hypotheses on the geographic mosaic of coevolution. Am Nat. 1999;153: S1–S14.

[66] Joppa LN, Roberts DL, Myers N, Pimm SL. Biodiversity hotspots house most undiscovered plant species. Proc Natl Acad Sci U.S.A. 2011;108: 13171–13176. doi: 10.1073/pnas.1109389108 21730155 10.1073/pnas.1109389108 PMC3156159

[67] Hamilton AJ, Basset Y, Benke KK, Grimbacher PS, Miller SE, Novotný V, et al Quantifying uncertainty in estimation of tropical arthropod species richness. Amer Nat. 2010;176: 90–95. 20455708 10.1086/652998

