# Supplementary material for: Hidden biodiversity in entomological collections: The overlooked co-occurrence of dipteran and hymenopteran ant parasitoids in stored biological material
Source: PLoS One. 2017 Sep 19;12(9):e0184614. doi: 10.1371/journal.pone.0184614 (PMC5604966; doi:10.1371/journal.pone.0184614)
Supplement: S1 Table — All colony samples were collected in 1999 from Zoh-Laguna, Calakmul (Campeche, Mexico) and stored in the Formicidae Collection (ECO-CH-F) of El Colegio de la Frontera Sur in Chetumal (Quintana Roo, Mexico). (PDF) [file pone.0184614.s001.pdf]

S1 Table. ***Neoponera villosa*** **colony samples used in this study.** All colony samples were collected in 1999 from Zoh-Laguna, Calakmul (Campeche, Mexico) and stored in the Formicidae Collection (ECO-CH-F) of El Colegio de la Frontera Sur in Chetumal (Quintana Roo, Mexico).

ECO-CH-F Code	Locality	Date of collection	Nest composition					
			Queens	Workers	Males	Gynes	Larvae	Pupae
F-0275	Campeche, Calakmul, 6 km N of Zoh-Laguna	April 21, 1999	0	22	0	0	7	0
F-0276	Campeche, Calakmul, 6 km N of Zoh-Laguna	April 21, 1999	0	15	0	0	0	2
F-0281	Campeche, Calakmul, 8km N - 2 km E of Zoh-Laguna, near Aguada	April 22, 1999	2	197	60	29	2	23
F-0287	Campeche, Calakmul, 8 km N - 4 km E of Zoh-Laguna	April 22, 1999	0	78	0	26	1	32
F-0301	Campeche, Calakmul, 12 km N - 2.5 km E of Zoh-Laguna	April 23, 1999	1	36	0	5	0	8
F-0303	Campeche, Calakmul, 12 km N - 2.5 E of Zoh-Laguna	April 23, 1999	0	102	9	8	2	25
F-0308	Calakmul, Calackmul, Camino a Aguada del Cocodrilo-Papagayo	April 24, 1999	5	55	1	0	0	7
F-0310	Calackmul, Calakmul, Camino a Aguada “Las Trampas”-Papagayo	April 24, 1999	1	47	0	0	0	13
F-0316	Calakmul, Calakmul, Aguada Abejas 24 km N of Zoh-Laguna Papagayo	April 24, 1999	0	60	0	0	0	2
