# Supplementary material for: Hidden biodiversity in entomological collections: The overlooked co-occurrence of dipteran and hymenopteran ant parasitoids in stored biological material
Source: PLoS One. 2017 Sep 19;12(9):e0184614. doi: 10.1371/journal.pone.0184614 (PMC5604966; doi:10.1371/journal.pone.0184614)

S1 Fig. A worker of the ant host *Neoponera villosa*. (A) Left profile of the worker showing the almost straight, vertical anterior face of the petiole, the posterior face broadly convex and the reddish base of the legs. (B) Close-up of the petiole. Photo: J-P Lachaud.

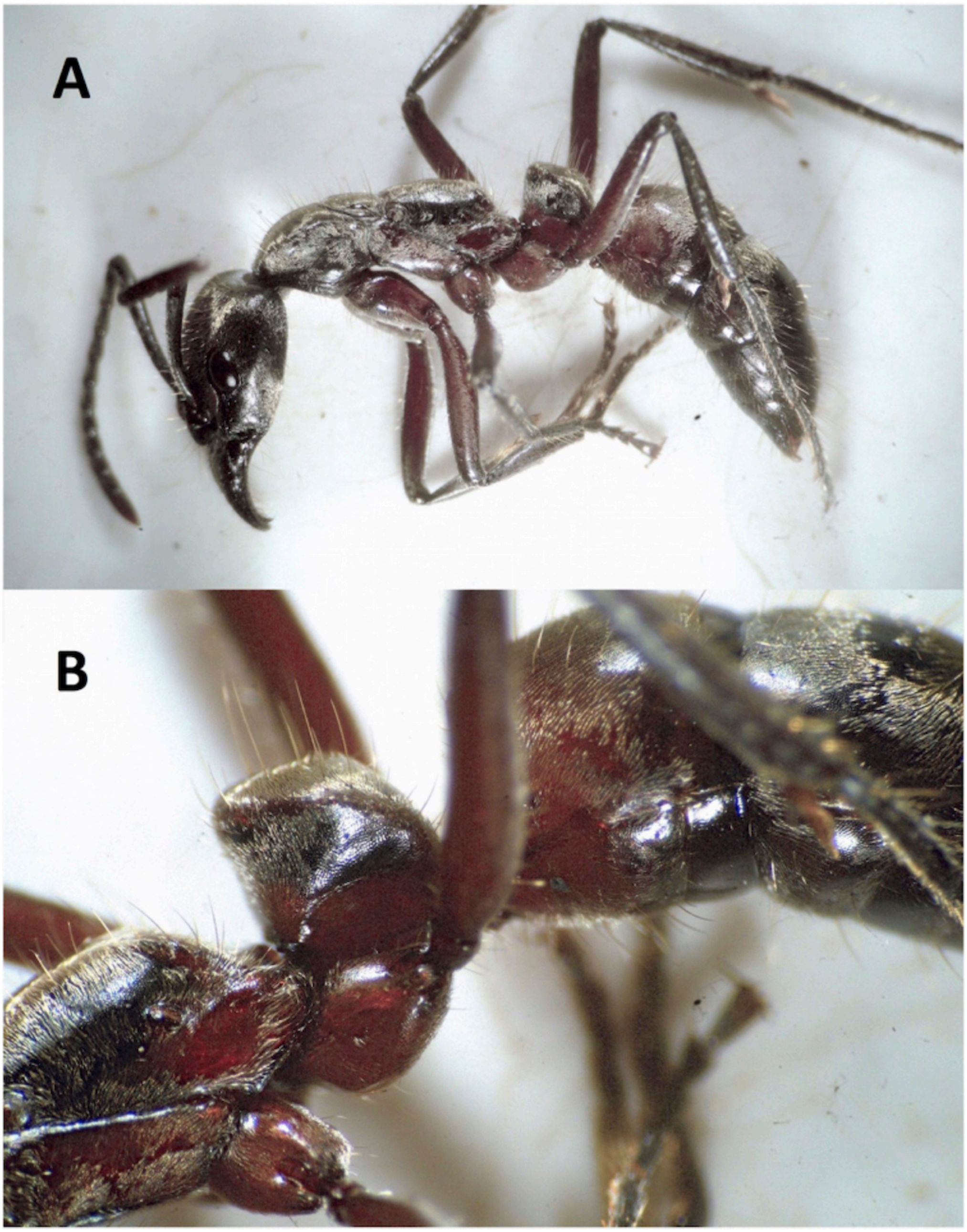

Supplement: S1 Fig — (A) Left profile of the worker showing the almost straight, vertical anterior face of the petiole, the posterior face broadly convex, and the reddish base of the legs. (B) Close-up of the petiole. Photo: J-P Lachaud. (PDF) [file pone.0184614.s002.pdf]
